# Large animal models enhance the study of crypt-mediated epithelial recovery from prolonged intestinal ischemia reperfusion injury

**DOI:** 10.1152/ajpgi.00236.2024

**Published:** 2024-10-15

**Authors:** Caroline A. McKinney-Aguirre, Cecilia R. Schaaf, Elizabeth Goya-Jorge, John M. Freund, Liara M. Gonzalez

**Affiliations:** Department of Clinical Sciences, North Carolina State University College of Veterinary Medicine, Raleigh, North Carolina, United States

**Keywords:** intestinal ischemia reperfusion injury, intestinal stem cell, mouse model, porcine model

## Abstract

Intestinal ischemia and reperfusion injury (IRI) is a deadly and common condition. Death is associated with sepsis due to insufficient epithelial repair, requiring stem cell-driven regeneration, typically beginning 48 h after injury. Animal models are critical to advancing this field. To effectively study epithelial healing, models must survive clinically relevant intestinal ischemic injury extending to the crypt. Although mouse models are indispensable to intestinal research, their application for studying epithelial repair following severe IRI may be limited. Ischemic injury was induced in mouse and porcine jejunum for up to 3 h, with up to 72 h of reperfusion. Histologic damage was scored by Chiu-Park grade, and animal survival was assessed. Findings were compared between species. A mouse IRI literature review was performed to evaluate the purported degree of injury, duration of recovery, and reported survival rates. In mice and pigs, 3 h of ischemia induced severe, reliable injury extending into the crypt. However, at 48 h, mouse survival was only 23.5% compared with 100% survival in pigs. In literature, ischemia was induced for >1 h in only 4 of 102 mouse studies and none to 3 h. Recovery was attempted for 48 h in only six reports. Forty-seven studies reported intestinal crypt injury. Of those that featured histologic intestinal crypt damage, survival rates at 48 h ranged from 10 to 50% (median 30%). Mouse models are not ideal for studying intestinal stem cell-mediated recovery from severe IRI. Alternative large animal models, like pigs, are recommended.

**NEW & NOTEWORTHY** Additional research is needed to improve recovery from severe intestinal ischemia. The selection of the ideal animal model is critical to facilitating this work. Based on our experimentation and literature review, porcine models, with increased translatability and an improved ability to survive both prolonged ischemia and the recovery period, appear to be the most appropriate choice for future studies.

## Graphical abstract

**Figure:**
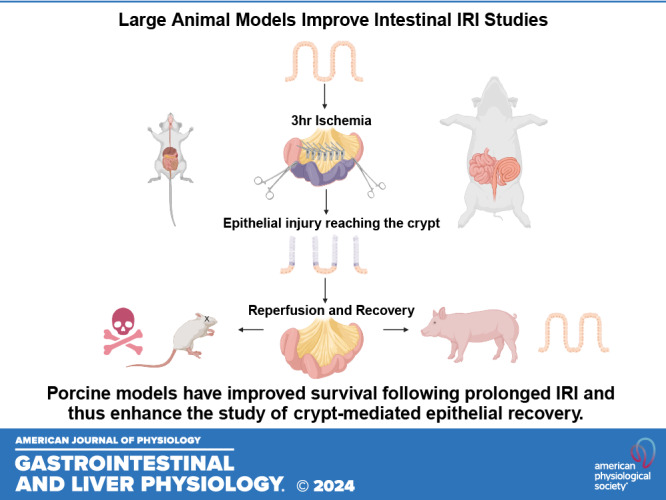

## INTRODUCTION

Intestinal ischemia and reperfusion injury (IRI) is a critical pathologic component of numerous disease states (1). IRI induces significant intestinal epithelial barrier compromise and permits bacterial translocation, potentially resulting in sepsis and shock. Acute mesenteric ischemia, for example, has a mortality rate approaching 80% (2). The inability to prevent these lesions and the challenge of early diagnosis contribute to disease severity. As such, patients face significant epithelial loss and rely upon inherent intestinal epithelial healing to prevent damaging sequelae. Although the small intestine is highly susceptible to IRI, crypt-base intestinal stem cells drive impressive epithelial recovery (3–5). Unfortunately, few studies induce clinically relevant ischemic injury that effectively recapitulates the severe epithelial loss seen in humans. Even fewer studies use adequate survival periods to examine tissue repair mechanisms. Ultimately, this has likely contributed to the limited development of clinically relevant therapeutics.

Animal models have been vital to elucidate mechanisms of ischemic injury and recovery, and rodents are the most employed. However, key anatomic and physiologic intestinal dissimilarities between humans and rodents limit their translational capacity (1, 6). Specifically, mice have significant differences in intestinal permeability, vascular anatomy, tissue enzymes, and hypoxic response pathways, which could impact the induction of and response to IRI (6–9). Overall, there is a greater anatomic and physiologic correlation between humans and pigs (7). Thus, the selection of the best animal model for the pathophysiology in question is of paramount importance.

Other attributes of experimental design can also limit the utility of IRI models. Most rodent studies, for example, only use short durations of ischemia, which do not reflect most human clinical timelines. In humans, less than 30 min of ischemia results in minor lifting of the villus tip epithelium from the basement membrane (Gruenhagen space), which can reseal within 60 min of reperfusion (10). Prolonged ischemia, up to 3 h, is more common in clinical patients and induces crypt-base injury equivalent to Chiu-Park grade 4 (3). Appropriate modeling of both prolonged ischemia (up to 3 h) and sufficient time for cellular proliferation (>12 h reperfusion) is necessary to understand intestinal crypt-driven recovery (11). Specifically, our previous work demonstrated that 48 h is required for early intestinal stem cell regeneration and epithelial recovery following prolonged IRI (4).

In this report, we examined the utility of intestinal repair experimentation in mouse and porcine models following prolonged ischemia. We focused on severe injury that both parallels clinical cases and impacts progenitor cells within the epithelial crypt base. To evaluate this, we performed experimental ischemia-reperfusion modeling to compare species-specific outcomes and reviewed mouse IRI literature. Through this approach, we highlighted the strengths and limitations of these IRI animal models.

## METHODS

The Institutional Animal Care and Use Committee (IACUC) of North Carolina State University approved all animal experimentation (mice: IACUC no. 20-475, *n* = 25, 8–15 wk old, 20–25 g, C57BL6; pigs: IACUC no. 21-491, *n* = 7, 8–10 wk old, 15–25 kg, Yorkshire cross). Mice and pigs of mixed sex were subjected to segmental mesenteric arterial occlusion for 30 min–3 h (3, 4, 12). Food was withheld for no longer than 18 h in some mice and all pigs (*n* = 15 mice; *n* = 7 pigs).

Mice were anesthetized using 5% vaporized isoflurane in 100% O_2_ followed by maintenance anesthesia at 1%–2% vaporized isoflurane via nose cone. Pigs were anesthetized following induction with xylazine [1.5 mg/kg intramuscularly (im)] and ketamine (11–20 mg/kg im). Orotracheal intubation was performed, and pigs were maintained under general anesthesia with isoflurane (2%–5%, in 100% O_2_). For mice, the anesthetic depth was monitored by withdrawal reflex and respiratory rate, whereas pigs were additionally monitored with jaw tone, palpebral response, and parameters including electrocardiography, pulse oximetry, and indirect blood pressure measurement. For both, the temperature was monitored and supported using a water-circulating heating mat and heat lamps. Mice were administered 1 mL of subcutaneous normothermic saline, which also provided hydration, whereas pigs received intravenous isotonic fluids (15 mL/kg/h). Abdomens were aseptically prepared with a surgical scrub (Chlorhexidine) and alcohol. A ventral midline skin incision (mice: 3 cm; pigs: 12 cm) was followed by an incision into the linea alba. In pigs, the jejunum was identified 40 cm oral to the ileocecal junction, whereas in mice, small intestine was isolated at the approximate midpoint between stomach and colon. Segments of intact intestines (mice: 2 cm; pigs: 10 cm) were carefully exteriorized and kept hydrated with saline. Loops were demarcated with luminal clamping (mice: Johns Hopkins Bulldog clamps; pigs: doyen forceps). Ischemia was induced by clamping vascular supply to a designated segment of the intestine using Johns Hopkins Bulldog clamps for up to 3 h. The intestinal segments were replaced into the abdominal cavity, skin temporarily closed with towel clamps and covered with saline-soaked gauze for the duration of ischemia.

For both species, after ischemia clamps were removed, intestines were replaced in the body cavity, and carboxymethylcellulose was added to reduce adhesion risk. In pigs, sterile saline abdominal lavage was also performed. The abdomen was closed in 2 (mice) or 3 (pig) layers and animals were recovered. During recovery, animals were monitored continuously until they regained the ability to stand and ambulate appropriately. Before ambulation, mice were treated with 0.01 mg/kg buprenorphine subcutaneously for pain. Additional pain medication (buprenorphine subcutaneously: mice 0.01 mg/kg; pigs 0.02–0.05 mg/kg) was administered as needed. Regular monitoring, every 6–12 h, was continued until the recovery end point, up to 72 h, or humane end point. Mice were euthanized via cervical dislocation following anesthesia using isoflurane. Pigs were euthanized with pentobarbital (85–100 mg/kg intravenously) following induction with xylazine and ketamine.

Tissue was collected at the time of euthanasia for histologic analysis of epithelial injury using Chiu-Park grading (Table 1) (13). A survival curve was determined for animals where recovery was planned or attempted.

**Table 1.** Chiu-Park grading applied to ischemically injured mouse and porcine tissue

Chiu-Park Score	Description of Tissue Damage
0	Normal mucosal villi
1	Formation of subepithelial Gruenhagen’s space at villus apex; capillary congestion
2	Subepithelial space extension with moderate lifting of epithelial layer from the lamina propria
3	Massive epithelial lifting down the sides of the villi; a few villus tips may be denuded
4	Denuded villi exposing lamina propria and dilated capillaries; increased cellularity of lamina propria may be noted
5	Lamina propria is digested and disintegrated; hemorrhage and ulceration are present

To evaluate current practices and durations of ischemia and reperfusion reported in the field, over 100 original research articles utilizing this methodology were reviewed. Articles in PubMed (2001–2024) were identified using Boolean operators (AND and OR) to combine terms such as intestinal, ischemia, reperfusion, and mouse. Relevant articles were identified through title and abstract screening, followed by full-text assessment.

## RESULTS AND DISCUSSION

### Results

#### Mouse and porcine ischemia-reperfusion experiments.

**Figure 1.**
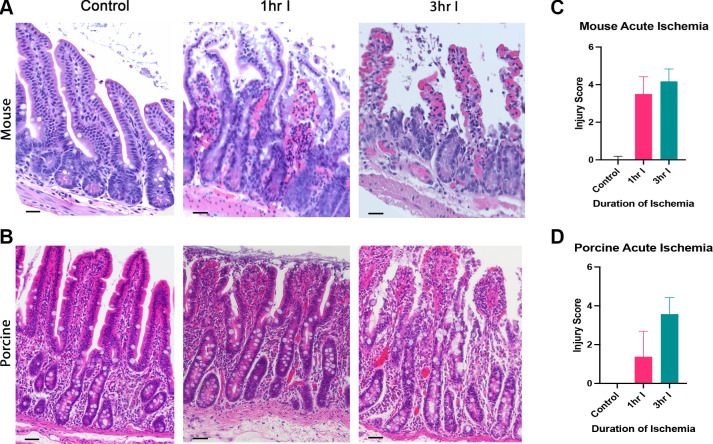
Prolonged ischemia is required to damage the epithelium to the level of the crypt. Representative images of jejunum following no ischemia (control), 1 h of ischemia (1 h I), and 3 h of ischemia (3 h I) in mice (*A*) and pigs (*B*). Scale bar = 50 µm, all images taken at ×20. Graphical representation of Chiu-Park injury scores assigned after injury for mice (*C*, *n* = 4) and pigs (*D*, *n* = 4–7).

A time-dependent, progressive loss of epithelium beginning from the villus tip and extending toward the crypt was observed, as previously described in mouse, porcine, and human injury (1, 3, 12). Crypt damage was observed by 3 h of ischemia, but not prior, in both species (Fig. 1).

**Figure 2.**
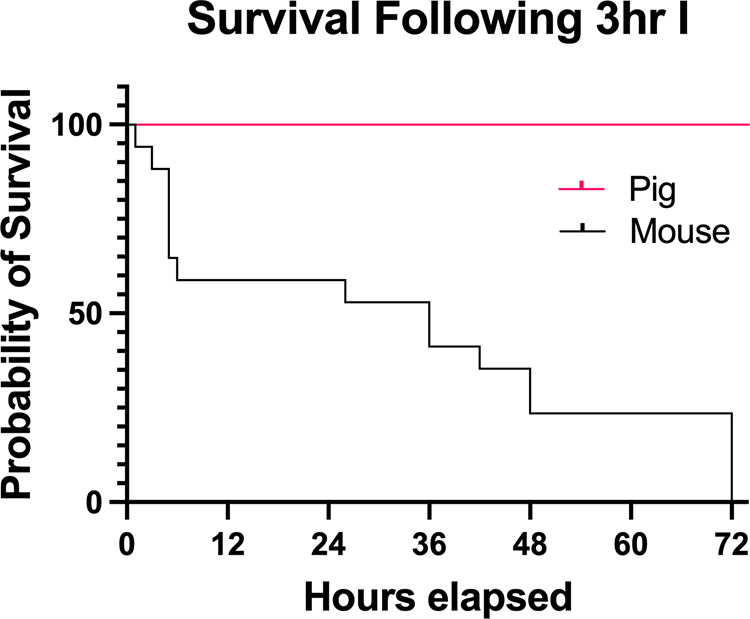
Mouse survival after prolonged ischemia is insufficient to study epithelial recovery. Kaplan–Meier survival prediction comparing mice and pigs following 3 h I.

The mouse mortality rate during 3 h of ischemia (3 h I) was 4.2%. However, in the 12 h following, 41.2% of remaining mice were found dead or required emergency euthanasia, increasing to 52.9% at 24 h. By 48 h, 76.5% had died and, by 72 h, mortality increased to 84.6%. In contrast, all pigs who underwent 3 h I survived to 72 h (Fig. 2).

**Figure 3.**
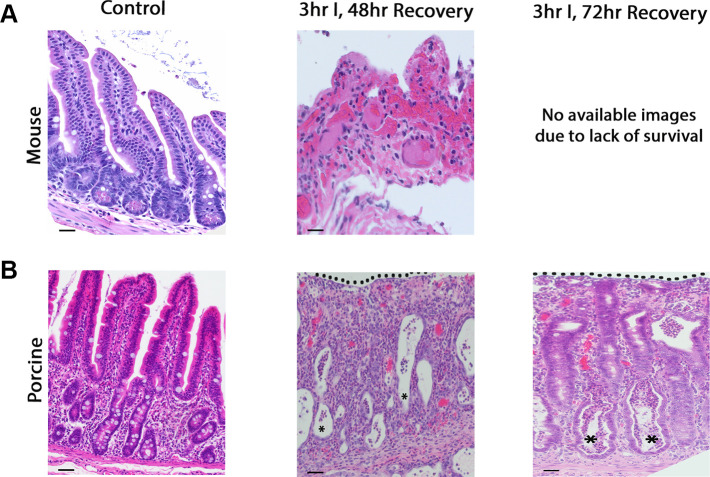
Mouse intestinal epithelium does not show productive recovery following prolonged ischemia. Representative images of jejunum following no ischemia (control), 48 h following 3 h ischemia in mice (*A*) and porcine tissue (*B*) (3 h I, 48 h recovery), and 72 h following 3 h ischemia in porcine (*B*). Dotted lines indicate re-epithelialization and asterisks indicate crypt regeneration. Scale bar = 50 µm, all images taken at ×20.

Furthermore, mice that survived until 48 h following 3 h I showed no histological evidence of recovery-associated crypt depth expansion or re-epithelialization, contrasting such reparative events observed in pigs (Fig. 3). In pigs, evidence of progressive repair was observed at 72 h of recovery (Fig. 3*B*). Because of lack of survival, representative mouse imaging is not available. All mice who underwent only 30 min of ischemia survived until 48 h (data not shown).

#### Evaluation of published mouse ischemia-reperfusion experiments.

One hundred and two articles were reviewed based on their application of mouse intestinal IRI (Supplementary Table 1: https://dx.doi.org/10.6084/m9.figshare.27190248). Only 4 of 102 articles extended ischemia beyond 1 h, and none continued ischemia beyond 90 min. All studies employed superior mesenteric artery clamping, compared with the segmental ischemia used here. Durations of ischemia and reperfusion reported by each study are highlighted in the table. Of those reporting injury scores, 47 of 102 reported damage equivalent to a Chiu-Park score of 4 or higher, consistent with epithelial damage to the level of the intestinal crypt. For those who reported this degree of damage and euthanasia endpoints, 16 of 47 attempted recovery for 12 h, whereas 15 of 47 did so for 24 h, and only 6 of 47 for 48 h, with all others euthanizing mice several hours following ischemia. Within those attempting 12 h of recovery, 8 of 16 papers reported survival rates, which ranged from 25 to 100% (mean 72.5%). Of 15, 7 papers tracked recovery to 24 h, which ranged from 0 to 100% (mean 49.38%), and 4 of 6 of those recovering for 48 h recorded survival rates of 0%–50% (mean 31.0%). Although 47 papers reported injury scores of a Chiu-Park score of 4 or higher, our re-evaluation of their representative images found that only 3 reports featured images consistent with this degree of injury. Within these three reports, two reported survival rates at 12, 24, and 48 h, which ranged from 75 to 100% (mean 100%), 10%–75% (mean 60%), and 10%–50% (mean 30%), respectively.

## DISCUSSION

Mouse models are an invaluable asset to research; however, in severe IRI studies, they have significant limitations. From our experimentation, severe IRI and survival cannot be reliably studied in mice, as compared with pigs. In addition, we summarized similar limitations in other IRI mouse model papers. Our findings suggest that large animal models, specifically porcine, provide a more reliable and translational framework to study mechanisms of and advance therapies for severe IRI.

From a technical perspective, severe injury (3 h ischemia) can be induced in mice; however, subsequent survival, required to evaluate stem-cell mediated recovery, is less attainable. The challenges of recovering mice beyond severe IRI are evidenced, in part, by the lack of prolonged recovery attempted in the literature, with only 16 of 47 papers attempting recovery for 12 h. As El-Assal and colleagues (14) remarked, their initial ischemia modeling “showed that 90 min of ischemia resulted in over 70% mortality,” which precludes sufficient data collection to draw conclusions. Our attempts to increase survival through segmental vascular occlusion, which reduces the total length of the ischemic intestine compared with the more commonly employed superior mesenteric artery occlusion, did not improve mortality rates and instead was associated with intestinal distension. This approach was utilized, in part, to reduce the proportion of experimentally manipulated intestines compared with both the entire intestinal tract and animal size. Given the smaller size of mice, inducing injury in only 2 cm of the total small intestine represents a significant proportion of tissue damage and increased risks of mortality (2, 15). As pigs and humans have more comparable total lengths of small intestine, this factor is less of a confounding variable (1, 16). The increased length of the porcine intestine also allows for the creation of discrete sections of the intestine that can be isolated to induce different degrees of injury or introduce local therapeutics, with each animal serving as an internal control (1, 3, 4, 12).

Animal size also impacts the extent of feasible surgical monitoring and supportive care. Here, both species were managed with constant surveillance during the peri- and immediate postoperative periods and treated with standard of care following recovery. Because of their smaller size, intravenous fluid resuscitation is nearly impossible in mice and thus only subcutaneous fluids were administered (17). In addition, murine endotracheal administration is very challenging and thus nose cone-delivered inhalant anesthesia was used. Both species were treated with the same analgesic agent, buprenorphine, following surgery, though pigs’ approved dosing ranges extended higher. Until equipment is available to better facilitate murine endotracheal intubation, blood pressure monitoring, and intravenous fluids and medication administration, these challenges will persist, thus highlighting another benefit to working with larger species. Within the confines of available technologies, efforts were taken to provide comprehensive care and monitoring; however, in the future, more standardized protocols may result in improved survival in mice. Beyond procedural difficulties, injury evaluation methodology is also limited. The most employed scoring method, Chiu-Park, is not ideal for evaluating tissue during reperfusion, requiring researchers to develop independent systems making comparisons between studies challenging (4). The inclusion of additional markers of tissue injury and apoptosis, such as Cleaved Caspase 3 or intestinal fatty acid binding protein, and markers of stem cell-mediated regeneration including sex-determining region Y-box 9 and Ki67 colocalization or olfactomedin 4, would expand future model comparisons.

Mortality, though a simplistic representation of experimental success, was evaluated here as it ultimately determines the degree of recovery that can be studied. It is critical to acknowledge, however, the many biological responses contributing to mortality. These include differences in anatomy, baseline physiology, and inflammatory responses. Direct comparisons to clinically relevant human IRI are complicated by the paucity of human tissue samples with controlled durations of injury and recovery. One group successfully created a model of human small intestine IRI and demonstrated that after brief periods of ischemia (<1 h) epithelial loss is present only in the apical villi, as observed in mouse and pig models (10, 18). To our knowledge, longer durations of human IRI have not been experimentally explored.

In comparison to humans, and pigs, mice have reduced vascular arborization and venules, which directly impacts sensitivity to ischemic injury (19–21). Rodents also have increased intestinal mucosal oxidase, which contributes to reperfusion injury exceeding that seen in humans with similar degrees of ischemia (6). Increased xanthine oxidase triggers initial neutrophilic infiltration and tissue inflammation, representing another difference between humans, pigs, and mice (1, 16). Beyond intestinal-specific differences, mice have an overall higher metabolic rate compared with pigs, who are much more comparable to humans (17, 22, 23). Reductions in available oxygen during ischemia may lead to more significant disturbances in energy metabolism, which may exacerbate intestinal injury experienced by mice during identical durations of ischemia in pigs and humans (24). As a further nuance of mouse models, they also have the unique capacity to swiftly and extensively decrease their basal metabolic rate, which further complicates interpretations of IRI responses (17). Overall, porcine subjects appear to offer a superior option for IRI modeling.

Porcine subjects are not the only large animals that have been evaluated for their utility in IRI modeling. Other species include cats and dogs. Cats, however, like rodents, have increased xanthine oxidase and larger populations of local inflammatory cells, such as neutrophils, which contribute to increased reperfusion injury as compared with humans and pigs (1, 25, 26). Dogs have similar intestinal anatomy and physiology to humans; however, they are exquisitely sensitive to IRI and experience high associated mortality rates (27). The use of cats and dogs is also complicated by growing social aversion to companion animal species use in research (28). Mice, with their easy and inexpensive husbandry and established genetic manipulation tools, will continue to be an indispensable tool for some elements of IRI inquiry, especially that of shorter durations. Specifically, genetic tractability allows for the elimination and/or reduction of cell types in and surrounding the intestinal crypt, which could elucidate the roles of different populations in damage and repair. In addition, mice will remain a useful early in vivo model for exploring potential IRI dynamics prior to moving to more translatable models like the pig.

Selection of appropriate animal models is necessary to ensure accurate injury representation, translate to species of interest, and reduce and refine research animal use. Although mice will remain irreplaceable in the overall study of intestinal epithelial biology, their inability to model prolonged intestinal ischemia and recovery limits their translatability to humans experiencing this very clinically relevant injury. As porcine intestinal anatomy and physiology are inherently more human-like, their size allows multiple replicates and injury grades in one animal, and they survive the injury and recovery consistent with severe IRI in humans, we advocate that they are the ideal model for exploring mechanisms of healing following this injury.

## DATA AVAILABILITY

Data will be made available upon reasonable request.

## SUPPLEMENTAL MATERIAL

10.6084/m9.figshare.27190248 Supplemental Table 1: https://dx.doi.org/10.6084/m9.figshare.27190248.

## GRANTS

This study was supported by National Institutes of Health Grants NIH T32OD011130-15 (to C.A.M.A.), K01OD010199 SERCA, R03 OD026598-01 (to L.M.G.), and P30 DK034987; and by United States Department of Defense Grants W81XWH-19-1-0677 (to L.M.G.) and W81XWH-19-1-0676.

## DISCLOSURES

No conflicts of interest, financial or otherwise, are declared by the authors.

## AUTHOR CONTRIBUTIONS

C.A.M.A., C.R.S., and L.M.G. conceived and designed research; C.A.M.A., C.R.S., J.M.F., and L.M.G. performed experiments; C.A.M.A. and E.G.J. analyzed data; C.A.M.A., E.G.J., and L.M.G. interpreted results of experiments; C.A.M.A. prepared figures; C.A.M.A. drafted manuscript; C.A.M.A., C.R.S., E.G.J., and L.M.G. edited and revised manuscript; C.A.M.A., C.R.S., J.M.F., and L.M.G. approved final version of manuscript.
